# FRI-1 Is an Anti-Cancer Isoquinolinequinone That Inhibits the Mitochondrial Bioenergetics and Blocks Metabolic Shifts by Redox Disruption in Breast Cancer Cells

**DOI:** 10.3390/antiox10101618

**Published:** 2021-10-14

**Authors:** Miguel Córdova-Delgado, Sebastián Fuentes-Retamal, Charlotte Palominos, Camila López-Torres, Daniela Guzmán-Rivera, Oney Ramírez-Rodríguez, Ramiro Araya-Maturana, Félix A. Urra

**Affiliations:** 1 Laboratorio de Plasticidad Metabólica y Bioenergética, Programa de Farmacología Molecular y Clínica, Instituto de Ciencias Biomédicas (ICBM), Facultad de Medicina, Universidad de Chile, Independencia 1027, Casilla 7, Santiago 8380453, Chile; cordovadelgado@ug.uchile.cl (M.C.-D.); sebastianfuentes@ug.uchile.cl (S.F.-R.); charlotte.palominos@ug.uchile.cl (C.P.); camila.lopez.t@ug.uchile.cl (C.L.-T.); 2 Network for Snake Venom Research and Drug Discovery, Santiago 7800003, Chile; 3 Escuela de Química y Farmacia, Facultad de Medicina, Universidad Andrés Bello, Santiago 8370149, Chile; daniela.guzman@unab.cl; 4 Laboratory of Chemistry and Biochemistry, Campus Lillo, University of Aysén, Eusebio Lillo 667, Coyhaique 5951537, Chile; oney.ramirez@uaysen.cl; 5 Instituto de Química de Recursos Naturales, Universidad de Talca, Casilla 747, Talca 3460000, Chile

**Keywords:** tricarboxylic acid (TCA) cycle, mitochondria, mitocans, 2-oxoglutarate dehydrogenase (OGDH), AMP-activated kinase (AMPK) signaling, CPI-613, devimistat

## Abstract

Since breast cancer (BC) cells are dependent on mitochondrial bioenergetics for promoting proliferation, survival, and metastasis, mitochondria highlight as an important target for anticancer drug discovery. FRI-1, methyl 1, 3-dimethyl-5, 8-dioxo-5, 8-dihydro-4-isoquinolinecarboxylate, was previously described as a selective cytotoxic compound on cancer cell lines, however, details on the mechanism of action remain unknown. In this work, we describe that FRI-1 inhibits mitochondrial bioenergetics, producing apoptosis in MCF7 and MDA-MB-231 BC cell lines. FRI-1 decreases the maximal oxygen consumption rate (OCR), Δψm, NADH, and ATP levels, with a notable increase of mitochondrial reactive oxygen species (ROS) production, promoting AMPK activation with pro-survival effects. Moreover, FRI-1 inhibits the metabolic remodeling to glycolysis induced by oligomycin. In isolated tumoral mitochondria, FRI-1 increases Complex I and III-dependent OCR state 2, and this is sensitive to rotenone and antimycin A inhibitor additions, suggesting a redox cycling event. Remarkably, α-ketoglutarate and lipoic acid supplementation reversed and promoted, respectively, the FRI-1-induced apoptosis, suggesting that mitochondrial redox disruption affects 2-oxoglutarate dehydrogenase (OGDH) activity, and this is involved in their anticancer mechanism. Consistent with this, the combination of FRI-1 and CPI-613, a dual inhibitor of redox-sensible tricarboxylic acid (TCA) cycle enzymes PDH and OGDH, produced extensive BC cell death. Taken together, our results suggest that FRI-1 exhibits anticancer effects through inhibition of mitochondrial bioenergetics by redox disruption in BC cells.

## 1. Introduction

Quinones have a key role in electron transport in metabolic pathways and some biological oxidation reactions. Because of that, they are recognized as privileged scaffolds in medicinal chemistry, exhibiting a broad spectrum of pharmacological activities. Since they have good drug-like properties, quinone moiety-containing compounds have been useful as lead compounds for obtaining new active compounds [1]. Some of them act by generating reactive oxygen species (ROS), DNA intercalation, and bioreductive alkylation of biomolecules.

**Figure 1:**
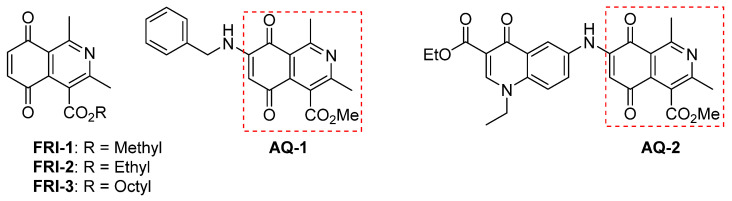
Chemical structures of isoquinolinequinones with biological effects reported.

FRI-2 (ethyl 1,3-dimethyl-5,8-dioxo-5,8-dihydroisoquinoline-4-carboxylate) is an isoquinolinequinone, reported about fifty years ago [2], and the synthesis of the methyl ester analog (FRI-1), was later improved (Figure 1). Their arylamino derivatives have attracted interest because of their antitumor effects on AGS human gastric adenocarcinoma cells (CRL-1739), HL-60 human leukemia cells (CCL-240), SK-MES-1 human lung cancer cells (HTB-58), and J82 human bladder carcinoma cells (HTB-1) [3], and antifungal and antibacterial activities [4,5]. Besides, recently has been reported the antitumor activities of AQ-1 against resistant breast cancer (BC) cell line MDA-MB 231 [6], among others. Another analog, AQ-2, also shows selective cytotoxicity on this metastatic BC cell line by activating AMP-activated kinase (AMPK), downregulating unfolded protein response pathway, and inhibiting mTOR signaling [7]. Although AMPK is an energy-sensing enzyme that is activated under energetic crisis and increased ROS production, coordinating cell growth, autophagy, metabolism, and drug response [8,9], the role of the AMPK activation and mitochondrial metabolism in the anticancer action of isoquinolinequinone pharmacophore remain uncertain.

Breast cancer is a heterogeneous disease comprised of several biologically distinct subtypes that have variations in the presence of estrogen receptors (ER), progesterone receptors (PR), and human epidermal growth factor receptor-2 (HER2). Of them, close to 60% are BC ER-positive and about 20% are negative for ER, PR, and HER2 expression [10]. Despite great progress in the early detection and development of clinical therapy, still is the leading cause of women’s death mainly associated with cancer metastases [11,12]. This highlights the need to explore new therapeutic approaches in BC.

A complex metabolic reprogramming during breast tumorigenesis determines the tumoral abilities for supporting proliferation, drug resistance, and metastasis [13,14,15,16]. Additionally, BC exhibits inter-tumor metabolic heterogeneity, where different BC types have a distinct and preferential metabolic phenotype [16,17]. Some evidence suggests that BC cells have an increasing dependence on oxidative phosphorylation (OXPHOS) during invasion and metastasis [18,19]. Although initial observations have suggested that tumor cells have high glycolysis with a reduced rate of mitochondrial oxygen consumption (known as the Warburg effect) [20], other non-energetic functions of the tumoral mitochondrion are currently recognized [21,22]. In fact, under hypoxia or electron transport chain inhibition, ATP levels are maintained by glycolysis, and NADH and lipidic precursors for proliferation are obtained from reductive carboxylation in a truncated TCA cycle dependent on 2-oxoglutarate dehydrogenase (OGDH) [23,24]. This is a Ca^2+^-dependent TCA cycle enzyme composed of three subunits (E1, E2, and E3) [25], that uses lipoic acid as a cofactor and catalyzes 2-oxoglutarate conversion to succinyl-CoA. OGDH is essential for maintaining Complex I-dependent mitochondrial respiration by control the mitochondrial NAD^+^/NADH ratio [26,27]. Since the lipoylated E2 subunits are present in OGDH and PDH, both enzymes are sensitive to inhibition by mitochondrial redox imbalance [28]. Several small molecules inhibit the mitochondrial bioenergetics, by inhibiting mitochondrial electron transport and/or by uncoupling of OXPHOS, with different consequences on viability and migration of cancer cells [29,30,31]. In particular, the induction of apoptotic cell death is obtained by a mitochondrial ROS production-dependent mechanism [32] that is mediated by the interaction between small molecules and respiratory complexes. This becomes the mitochondrion an attractive target for small molecules that exhibit anticancer effects by mitochondrial ROS production.

In this work, we describe that FRI-1 inhibits mitochondrial bioenergetics by redox disruption, being an essential event in the apoptotic death induced in MCF7 and MDA-MB-231 breast cancer cell lines.

## 2. Materials and Methods

### 2.1. Chemical and Reagents

All reagents were obtained from Sigma-Aldrich Corp. (St. Louis, MO, USA). FRI-1 was synthesized according to previously described procedures [3]. Stock solutions of FRI-1 and FRI-3 were prepared in dimethyl sulfoxide (DMSO) and maintained at −20 °C.

### 2.2. Chemistry

General methods. ^1^H and ^13^C NMR spectra were obtained from a spectrometer operating at either 300.13 MHz (^1^H) or 75.47 MHz (^13^C). Chemical shifts are reported as ppm downfield from TMS for ^1^H NMR and relative to the central CDCl_3_ resonance (77.0 ppm) for ^13^C NMR. Melting points were obtained with a Kofler hot-stage apparatus and were not corrected. Infrared spectra were recorded with a NICOLET 510P FT-IR spectrophotometer (Thermo Fisher Scientific, Waltham, MA, USA). High-resolution mass spectra were obtained on a MAT 95XP Thermo Finnigan spectrometer (Thermo Fisher Scientific, Waltham, MA, USA). Commercially available starting materials and solvents were used without further purification. Silica gel 60 (70–230 mesh) and alu-Foil 60 F254 were used for column chromatography and analytical TLC, respectively.

#### 2.2.1. *Synthesis of Octyl and Methyl 1,3-Dimethyl-5,8-dioxo-5,8-dihydroisoquinoline-4-carboxylates* (FRI-1 and FRI-3)

The already reported methodology of obtention isoquinolinequinone carboxylate FRI-1 [3] was slightly changed as follows. A suspension of 2, 5-dihydroxyacetophenone (152 mg, 1.0 mmol), silver oxide (488 mg, 2.2 mmol), and magnesium sulfate (1 g) in dichloromethane (25 mL) was stirred for 1 h. Silver oxide (488 mg, 2.2 mmol) and alkyl (^2^Z)-3-amino-2-butenoate (1.0 mmol) were added to the mixture and the stirring continued for 90 min. The alternative mixture was filtered, and the solvent was removed to yield the corresponding crude quinone FRI-1 or FRI-3. The crude product was purified by column chromatography on flash silica gel using dichloromethane/ethyl acetate as eluent.

#### 2.2.2. *Synthesis of Octyl 1,3-Dimethyl-5,8-dioxo-5,8-dihydroisoquinoline-4-carboxylate* (FRI-3)

Follow the general method, using 304 mg (2 mmol) of 2, 5-dihydroxyacetophenone, 1.95 g (8.8 mmol) of Ag_2_O, and 427 mg (2 mmol) of octyl (^2^Z)-3-amino-2-butenoate, obtained according to Suárez, et al. [33], by an enzymatic synthesis reaction using *Candida antarctica* lipase B (CALB), column flash chromatography on silica gel and dichloromethane: ethyl acetate (32:1) as eluent allowed the isolation of FRI-3 (yellow solid, 75% yield);. MP: 43–45 °C IR (KBr, cm^−1^): 3030, 2954, 2921, 2869, 2852, 1726, 1680, 1664, 1569, 1542, 1469, 1379, 1327, 1290, 1249, 1211, 1119, 1090, 868, 724. ^1^H-NMR (300 MHz, CDCl_3_) δ: 0.88 (t, *J* = 6.7 Hz, ^3^H, CH_3_), 1.28 (br s, ^8^H, 4 × CH_2_), 1.36 (m, ^2^H, CH_2_), 1.79 (p, *J*_1_ = 6.8 Hz, *J*_2_ = 7.3 Hz, ^2^H, CH_2_), 2.64 (s, ^3^H, CH_3_), 2.97 (s, ^3^H, CH_3_), 4.45 (t, *J* = 6.8 Hz, ^2^H, CH_2_–O), 6.97 (s, ^2^H, H–C=). ^13^C-NMR (75 MHz, CDCl_3_) d: 14.05, 22.57, 22.77, 25.75, 25.85, 28.22, 29.08, 29.12, 31.70, 66.41 (CH_2_–O), 120.50, 124.83, 135.22, 136.60, 140.26, 160.08, 161.00, 168.10 (O=C–O), 184.11 (C=O), 185.13 (C=O). HRMS (EI, 70 eV), M^+^ m/z: calcd for C_20_H_25_NO_4_: 343.1784 found 343.1779.

### 2.3. Breast Cell Lines

Cell lines MCF-7 and MDA-MB-231 BC cells lines and MCF-10A non-tumorigenic mammary epithelial cell lines were purchased from the American Type Culture Collection (ATCC, Manassas, VA, USA). MCF-7 and MDA-MB-231 were maintained in high glucose DMEM medium supplemented with 10% fetal bovine serum and penicillin 100 UI/mL and streptomycin 100 µg/mL. MCF-10A was maintained in DMEM/F12 medium supplemented with 5% fetal horse serum. Cells were grown at 37 °C in a humidified atmosphere of 5% CO_2_. All culture media were not supplemented with pyruvate.

### 2.4. Measurement of Mitochondrial Membrane Potential (*Δ*ψm)

The Δψm was analyzed by flow cytometry, using tetramethylrhodamine methyl ester probe (TMRM, Molecular Probe, Eugene, OR, USA). MCF-7 or MDA-MB-231 cells (1.5 × 10^5^ cells/mL) were seeded into 24-well plates and were treated with DMSO (control) and compounds at the indicated concentrations for 4 h. Then the cells were washed with PBS and incubated with the TMRM probe (5 nM, non-quenching mode) for 20 min [31]. Then the cells were collected, washed and fluorescence was detected through flow cytometry. As a positive control, the uncoupling agent 200 nM CCCP was used.

### 2.5. Clonogenic Assay

MCF-7 and MDA-MB-231 cells (500 cells/well) were seeded in 6-well plates and allowed overnight to adhere. Next, FRI-1 (5, 10, and 25 µM) was added to wells for 24 h following which media was replaced with compound-free media. After 7 days of incubation, cells were washed with PBS, and colonies were stained with a crystal violet solution (0.5% *w*/*v*). A colony was considered for at least 50 cells [34]. The colonies were counted using the software ImageJ 1.43 m (NIH, Bethesda, MD, USA).

### 2.6. Cellular Respiration in Real-Time

Cellular oxygen consumption rate (OCR) was measured in real-time using an XFe96 Extracellular Flux Analyzer (Bioscience, Agilent, CA, USA). MCF7 and MDA-MB-231 BC cells (20,000 cells/well) were seeded on XFe96 V3-PS multiwell plates and kept overnight at 37 °C in 5% CO_2_ with culture medium containing glucose plus glutamine. On the next day, the culture medium was replaced with assay medium (unbuffered DMEM without phenol red and with 4 mM glutamine and 10 mM glucose, pH 7.4) 1 h before the assay. Mitochondrial function was evaluated using 1 µM oligomycin, 50 nM FCCP, 1 µM rotenone, and 1 µM antimycin A as we described [31]. The oxygen consumption rate (OCR) and extracellular acidification rate (ECAR) measurements were made with the specific excitation and emission wavelengths of the fluorescent probes for oxygen (532/650 nm) and protons (470/530 nm). Each experiment was performed in triplicate.

### 2.7. Respiration in Isolated Mitochondria

The mitochondrial suspension was prepared from TA3/Ha tumor cells according to [30]. Oxygen consumption rate (OCR) was monitored polarographically at 25 °C with a Clark electrode as described previously [35]. To determine the effect of FRI-1 on the OXPHOS, mitochondria were stimulated with substrates for respiratory chain complex I (500 mM glutamate + 500 mM malate), complex II (1.0 M succinate), complex III (250 mM duroquinol) and complex IV (100 mM TMPD + 1.0 M ascorbate) in a respiration medium that contains 200 mL sucrose, 50 mM KCl, 3 mM K_2_HPO_4_, 2 mM MgCl_2_, 0.5 mM EGTA, 3 mM HEPES, pH = 7.4. To evaluate the presence of cyanide-insensitive mitochondrial respiration and other mitochondrial inhibitors, isolated mitochondria were stimulated sequentially with glutamate plus malate (G + M, 500 mM), rotenone (ROT, 1 µM), antimycin A (AA,1 µM), and KCN (3 µM).

### 2.8. Mitochondrial ROS (mtROS) Levels

The mtROS levels were measured using staining with MitoSOX^®^ Red probe (Invitrogen, Carlsbad, CA, USA). MCF-7 and MDA-MB-231 (1.5 × 10^5^ cells/mL) were seeded into 12-well plates, incubated for 24 h, washed with PBS, and treated with vehicle (DMSO) and FRI-1 for 4 h. Next, cells were incubated with MitoSOX Red^®^ (5 µM) for 30 min. Then, they were recollected, washed and the fluorescence was detected by flow cytometry as described [31].

### 2.9. NAD(P)H and ATP Levels

The NAD(P)H levels were measured through the autofluorescence of these dinucleotides as described [31]. In brief, BC cells (1.5 × 10^5^ cells/mL) were seeded in 96-well plates and treated with vehicle (DMSO), 5 µM rotenone, and FRI-1 (10–25 µM) for 4 h. The autofluorescence was measured using an excitation wavelength of 340 nm and an emission of 428 nm. ATP levels were determined using a luciferin–luciferase assay system according to the specification of the CellTiter-Glo Luminescent Cell Viability Assay kit (Promega, WI, USA), as previously described [30]. In brief, MCF-7 and MDA-MB-231 (1.5 × 10^5^ cells/mL) were seeded into a 96-well plate and incubated with DMSO (control) or FRI-1 for 2 h. As a positive control, 1 µM oligomycin (ATP synthase inhibitor) and 1 µM rotenone (Complex I inhibitor) were used.

### 2.10. Cell Death Assay

Detection of cell death was performed with the Apoptosis/Necrosis Detection Kit (Abcam, Cambridge, UK) according to the manufacturer’s protocol. MCF-7 and MDA-MB-231 (2.0 × 10^5^ cells/mL) were seeded in a 12-well plate and allowed overnight to adhere. After 24 h of treatment with vehicle (DMSO) or FRI-1, cells were harvested, and Annexin V-FITC and PI were added to a final concentration of 2.5 mg/mL and 5 mg/mL, respectively. Annexin V-FITC and PI-labeled cells were analyzed by FACS (FACS canto, BD biosciences, San José, CA, USA) as we described [29].

### 2.11. MTT Assay

Changes in cell viability induced by FRI-1 were evaluated by MTT reduction assay [35]. Briefly, 1 × 10^4^ cells/well were plated in 96 well plates and incubated overnight. Next, the cells were incubated with increasing concentrations of FRI-1 (0, 5, 10, 25, and 50 μM), 1 µM rotenone or 1 µM antimycin A, respectively, for 48 h. Then, cells were washed with PBS, and incubated with MTT (0.5 mg/mL) for 1 h. Formazan crystals were solubilized using DMSO and absorbance values (570 nm) were obtained using an ELISA plate reader (BioRad, Hercules, CA, USA).

### 2.12. Western Blotting

5.0 × 10^5^ MCF7 and MDA-MB-231 cells were treated with DMSO (Control) or FRI-1 for 4 h and lysed on ice with RIPA buffer (Tris-Cl [50 mM], NaCl [150 mM], sodium dodecyl sulfate [SDS; 0.1%]) containing protease and phosphatase inhibitor cocktail (Cell Signaling Technology, Danvers, MA, USA). The protein concentrations were determined using a BCA Protein Assay Kit (Pierce, Rockford, IL, USA). 50 µg of protein samples were separated in 10% SDS-polyacrylamide gels and transferred to PDVF membranes (Millipore, Billerica, MA, USA). They were blocked in 5% fat-free milk for 1 h at room temperature and incubated overnight at 4 °C with the following primary antibodies: phospho-AMPKα rabbit (Thr172) (D79.5E, Cell Signaling, #4188, 1:1000), AMPKα rabbit (Cell Signaling, #2532, 1:1000), OGDH (E1W8H) (Cell Signaling #26865, 1:1000) and β-actin (C4) mouse (Santa Cruz Biotechnology, Dallas, TX, USA, sc-47778, 1:2000). Then followed by incubation for 2 h at room temperature with the secondary antibodies conjugated to horseradish peroxidase (HRP): anti-mouse m-IgG_K_ BP-HRP (Santa Cruz Biotechnology, sc-516102, 1:5000) or mouse anti-rabbit IgG-HRP (Santa Cruz Biotechnology, sc-2357, 1:5000), respectively. The membranes were exposed to the chemiluminescent reagent Luminata Forte Western HRP substrate (Millipore) and visualized using C-digit equipment (Li-Cor, Lincoln, NE, USA). The densitometric analysis was performed using ImageJ 1.47v software [31]. Uncropped Western blots are shown in Supplementary Information Figure S4.

### 2.13. Statistical Analysis

All statistical analyses were performed using Graph Pad Prism 5.0 (GraphPad Software, San Diego, CA, USA). Statistical analysis was performed using two-way ANOVA with Bonferroni’s post-test for pairwise comparisons. The data were considered statistically significant when *p* < 0.05.

## 3. Results

### 3.1. FRI-1 Inhibits Cell Proliferation, Clonogenic Capacity, and Induces Apoptosis in Breast Cancer Cells

**Figure 2:**
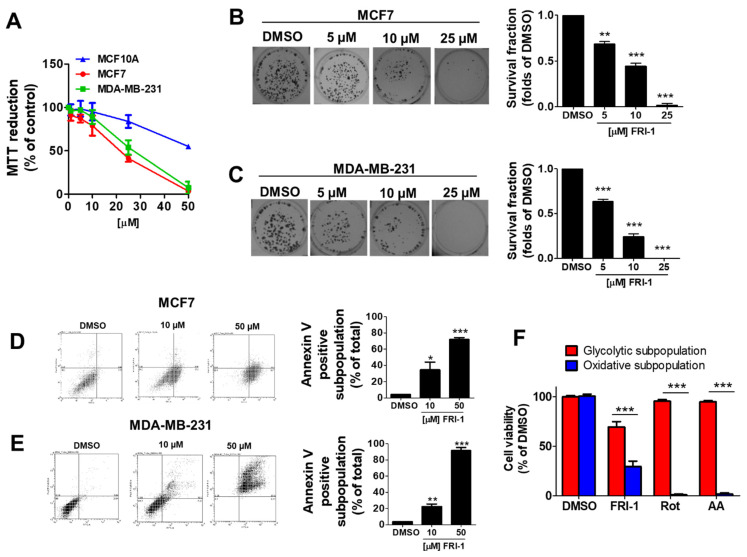
FRI-1 reduces the viability and clonogenic capacity of breast cancer cells. (**A**) Effect of FRI-1 on the viability of breast cancer cell lines (MCF7 and MDA-MB-231) and non-tumoral breast cell line (MCF10A) at 48 h of treatment measured by MTT assay; (**B**,**C**) Effect of FRI-1 on the clonogenic capacity of BC cells; (**D**,**E**) Determination of the percentage of apoptotic subpopulations in BC cells induced by FRI-1 at 48 h of treatment by Annexin V/PI assay; (**F**) Effect of FRI-1 (25 µM), rotenone (Rot, 1 µM) and antimycin A (AA, 1 µM) in glycolytic and oxidative subpopulations of MDA-MB-231 cells at 48 h of treatment. Viability was measured by MTT assay. The data shown are the mean ± SD of three independent experiments. * *p* < 0.05, ** *p* < 0.01, *** *p* < 0.001, vs. control (DMSO).

Consistent with previous reports [3], FRI-1 reduces selectively the viability in a concentration-dependent manner in MCF-7 and MDA-MB-231, with IC_50_ = 16.10 ± 1.39 µM and 21.93 ± 3.55 µM, respectively (Figure 2A). MCF10A cells resulted less sensitive to FRI-1 treatment (IC_50_ = 68.41 ± 6.27 µM) and significant effects on cell proliferation were only observed at higher concentrations, suggesting selectivity toward BC cells. Moreover, FRI-1 inhibits colony formation in a concentration-dependent manner in both BC cell lines (Figure 2B,C). To examine the type of BC cell death produced by FRI-1, flow cytometry analysis for Annexin V/PI was used. As shown in Figure 2D,E, FRI-1 increases annexin V-positive cells in both BC lines. To explore whether the effect of FRI-1 on cell viability is dependent on metabolism, we used two metabolically different subpopulations of MDA-MB-231 cells, which were generated by growing in glucose (25 mM) or galactose (10 mM) and glutamine (4 mM). In these conditions, the viability of glycolytic and highly oxidative MDA-MB-231 subpopulations are selectively sensitive to glycolytic or mitochondrial inhibitors as we previously reported [31]. At 48 h of treatment, Complex I and III inhibitors (1 µM rotenone and 1 µM antimycin A, respectively) induce cell death only in the oxidative subpopulation (Figure 2F). FRI-1 produces a significant reduction of viability in both subpopulations, which is higher in oxidative cells. This suggests that the anti-cancer effects of FRI-1 involve disruption of mitochondrial metabolism in BC cells.

### 3.2. FRI-1 Inhibits Mitochondrial Respiration and Blocks Metabolic Remodeling under Energetic Stress in Breast Cancer Cells

**Figure 3:**
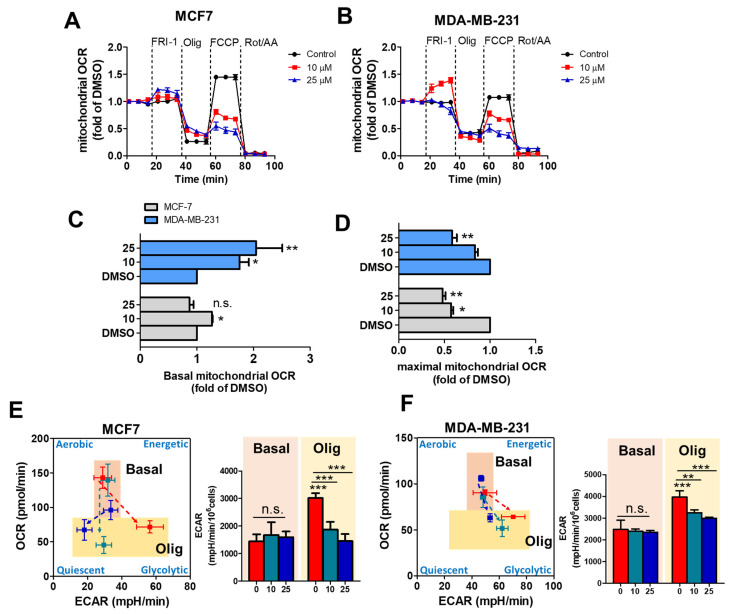
FRI-1 inhibits the metabolism of breast cancer cells. (**A**,**B**) Profile of mitochondrial respiration produced by FRI-1, oligomycin, FCCP, and rotenone/antimycin A injections; (**C**,**D**) Effect of FRI-1 on basal and maximal oxygen consumption rate (OCR) of BC cells; (**E**,**F**) Inhibition of oligomycin-induced metabolic remodeling by FRI-1 in MCF7 and MDA-MB-231 breast cancer cells. Data are shown as the mean ± SD of three independent experiments. * *p* < 0.05, ** *p* < 0.01, *** *p* < 0.001, vs. control (DMSO). n.s.: not significant.

We evaluate whether FRI-1 affects the mitochondrial bioenergetics in intact MCF7 and MDA-MB-231 cells lines. As Figure 3A,B shows, FRI-1 modifies the profile of mitochondrial respiration in BC cells, increasing the basal OCR (Figure 3C), and reducing the maximal OCR (Figure 3D). These effects are more pronounced in MDA-MB-231 cells. Since OXPHOS inhibition promotes a metabolic remodeling toward glycolysis as a compensatory response for maintaining ATP levels [36], we evaluate the effect of FRI-1 on extracellular acidification rate (ECAR) in basal condition and under oligomycin-induced metabolic stress. Notably, this compound does not produce a metabolic shift and it also inhibits the increase in glycolysis induced by oligomycin in both BC cell lines (Figure 3E,F). Taken together, these results suggest that FRI-1 reduces mitochondrial respiration, blocking the metabolic adaptations dependent on glycolysis.

### 3.3. FRI-1 Produces Inhibition of Mitochondrial Bioenergetics and Redox Stress in Breast Cancer Cells

**Figure 4:**
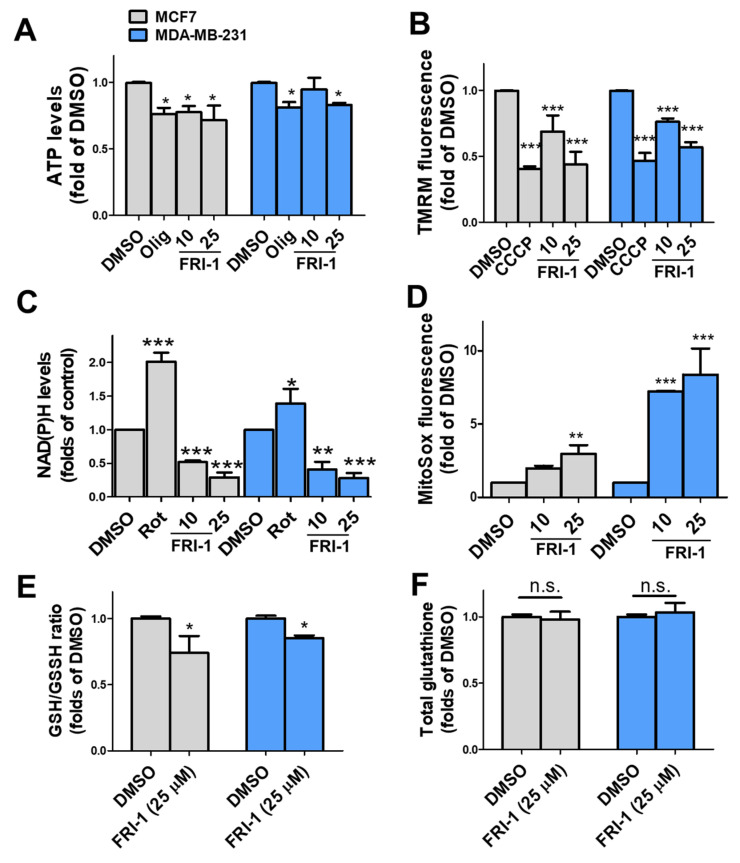
FRI-1 reduces mitochondrial bioenergetics, increasing mitochondrial ROS levels in breast cancer (BC) cells. (**A**) ATP levels; (**B**) Δψm, (**C**) NAD(P)H, (**D**) mitochondrial reactive oxygen species (ROS) levels, (**E**,**F**) GSH/GSSG and total glutathione levels at 4 h of treatment. BC cells were treated for 4 h and bioenergetic parameters were determined as described in the Section 2. Oligomycin (Olig, 1 µM), CCCP (200 nM), and rotenone (Rot, 1 µM). Data are shown as mean ± SD of three independent experiments. * *p* < 0.05, ** *p* < 0.01, *** *p* < 0.001, vs. control (DMSO). n.s.: Not significant.

We evaluated whether FRI-1-induced mitochondrial respiration alterations have consequences on bioenergetics of BC cells at 4 h of treatment. FRI-1 decreases the ATP and Δψm levels (Figure 4A,B). Consistent with the decreased NAD(P)H levels (Figure 4C), FRI-1 increases the mitochondrial superoxide production (Figure 4D), and it reduces the GSH/GSSG ratio (Figure 4E) without changes in the total glutathione (Figure 4F). Collectively, these results indicate that FRI-1 produces bioenergetics dysfunction and redox imbalance in BC cells.

### 3.4. FRI-1 Increases Oxygen Consumption Rate in Tumor Mitochondria

**Figure 5:**
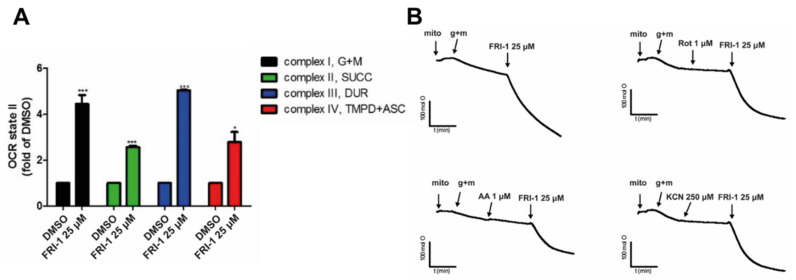
FRI-1 increases mitochondrial oxygen consumption rate in Complex I and III-dependent manners in isolated mitochondria. (**A**,**B**) Effect of FRI-1 on OCR in isolated mitochondria from murine BC cells in presence of respiratory substrates or complex inhibitors (Rot, AA and KCN). OCR was determined by Clark electrode. The data shown are the mean ± SD of three independent experiments. * *p* < 0.05, *** *p* < 0.001, vs. control (DMSO).

**Figure 6:**
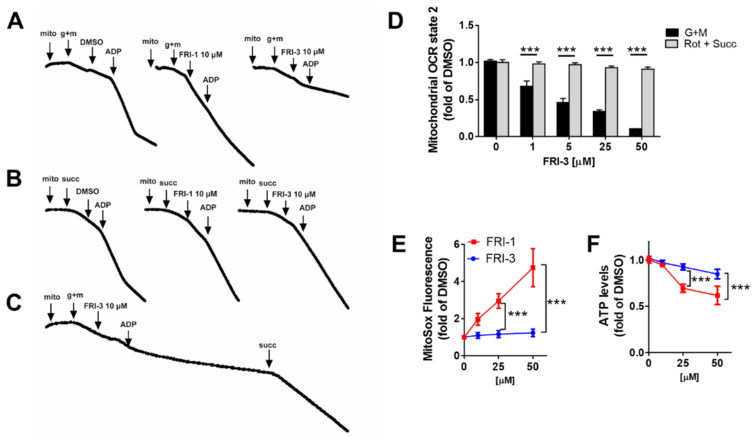
FRI-3 is an FRI-1 analog that acts on Complex I, lacking effects on ROS and ATP levels. (**A**–**D**) Effect of FRI-1 and FRI-3 on OCR in state 3. OCR was evaluated by Clark electrode; (**E**,**F**) Effect of FRI-1 and FRI-3 on the mitochondrial superoxide and ATP levels in MCF7 cells at 4 h of treatment. The data shown are the mean ± SD of three independent experiments. *** *p* < 0.001, vs. control (DMSO).

To determine whether the effect of FRI-1 on ROS production is related to interaction with respiratory complexes, we studied the effect of FRI-1 on mitochondria respiration using mitochondria isolated from a BC murine model. As Figure 5A shows, FRI-1 increases the OCR state 2_basal_ independently of the respiratory complex stimulated; however, the effect is greater when the complexes I and III had substrates. Interestingly, the total oxygen nanomoles consumed, but do not the OCR, during FRI-1-increased mitochondrial respiration, is sensitive to rotenone, antimycin A, and KCN inhibitors (Figure 5B). This explosive increase in the OCR of state 2_basal_ produced by FRI-1 no affects mostly the state 3_ADP_ (Figure 6A). To determine whether the redox behavior is dependent on the chemical modification of the FRI-1 structure, we obtained an FRI-1 analog containing an alkyl chain with eight carbons (FRI-3, Figure 6B,D), and the effects on mitochondrial respiration and bioenergetics were compared with FRI-1. FRI-3 inhibits the mitochondrial respiration dependent on Complex I without effects on Complex II. In contrast to the parental compound, FRI-3 lacks effects on ROS and ATP levels in MCF7 cancer cells (Figure 6E,F). Interestingly, FRI-3 inhibits the mitochondrial respiration in intact cells and promotes metabolic remodeling toward glycolysis, similarly to oligomycin, in BC cells (Supplementary information Figure S1). These results suggest that FRI-1 interferes with NADH and ubiquinol oxidation at Complex I and III, possibly by a mechanism of redox cycling, which may reduce the metabolic adaptations in BC cells.

### 3.5. FRI-1 Induces a Pro-Survival AMPK Signaling in BC Cells

**Figure 7:**
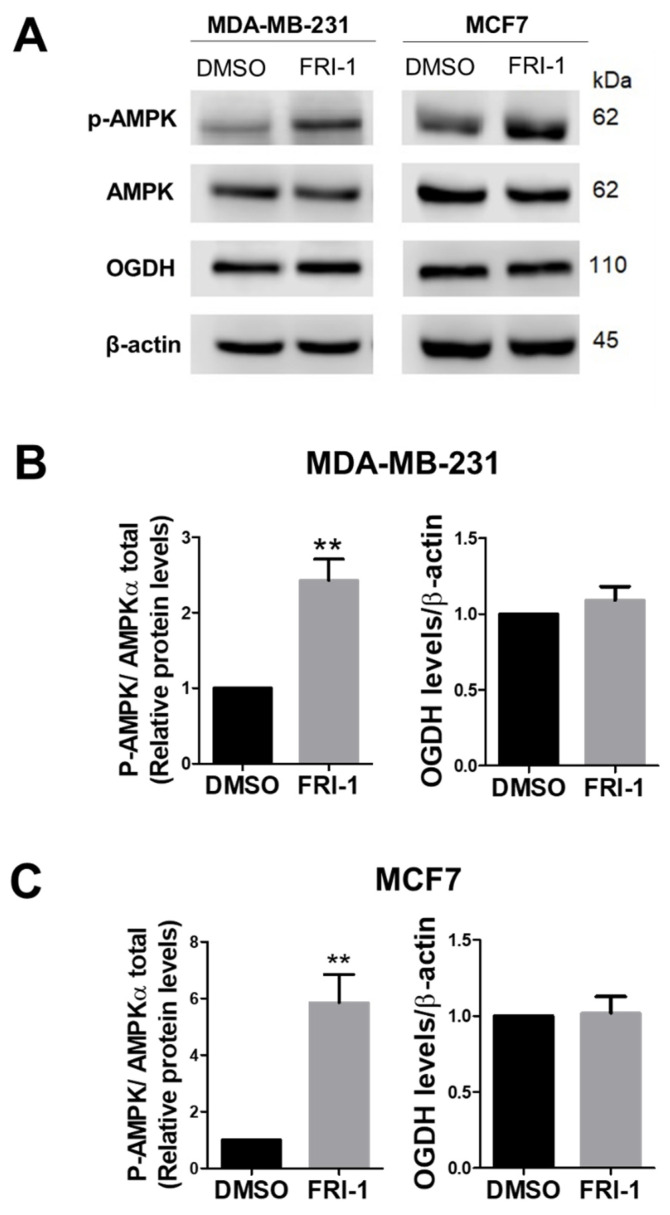
FRI-1 induces AMPK phosphorylation without changes in 2-oxoglutarate dehydrogenase (OGDH) levels in BC cells. (**A**) Representative figure of the effect of FRI-1 on phospho- and total-AMPK and OGDH levels at 4 h of treatment; (**B**) Quantification for MDA-MB-231 and (**C**) MCF7 cells. The data shown are the mean ± SD of three independent experiments. ** *p* < 0.01 vs. control (DMSO).

**Figure 8:**
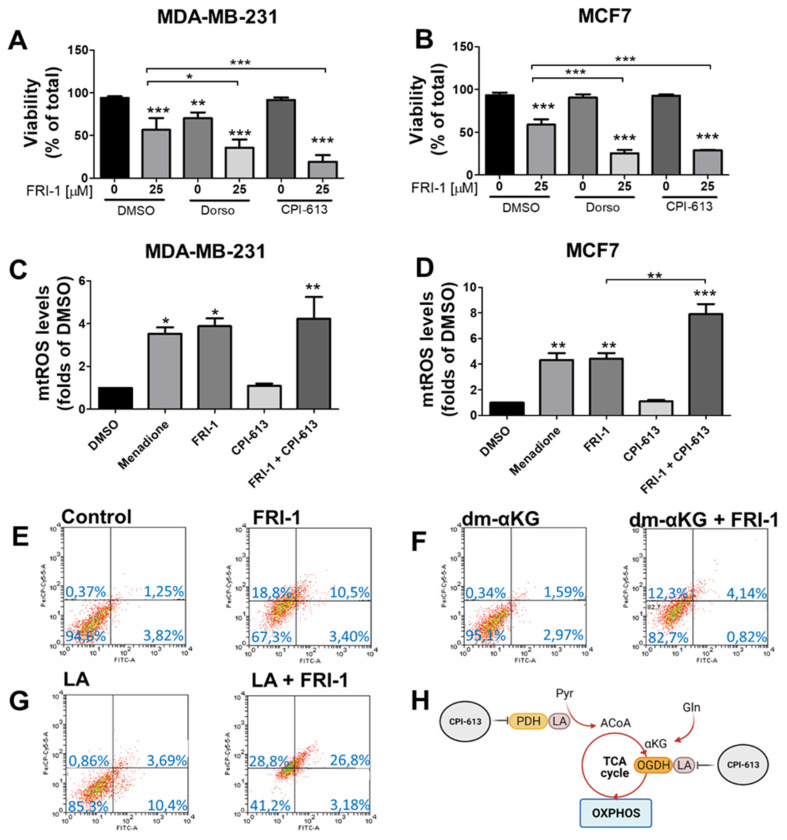
FRI-1 induces cell death by redox disruption of mitochondrial bioenergetics in breast cancer cells. (**A**,**B**) Effect of AMPK inhibition by dorsomorphin (Dorso, 10 µM) and CPI-613 (150 µM), dual inhibitor of PDH and αKGDH enzymes, on FRI-1-induced cell death at 48 h of treatment. (**C**,**D**) Effect of FRI-1 plus CPI-613 combination on mitochondrial ROS levels at 4 h of treatment in BC cells. (**E**–**G**) Effect of dimethyl-α-ketoglutarate (dm-αKG, 5 mM) and lipoic acid (LA, 5 mM) on FRI-1-induced MCF7 cell death at 48 h of treatment. (**H**) TCA cycle representation with sites of action of CPI-613. The data shown are the mean ± SD of three independent experiments. * *p* < 0.05, ** *p* < 0.01, *** *p* < 0.001, vs. control (DMSO). Abbreviations: LA: lipoic acid, ACoA; Acetyl-CoA, αKG: α-ketoglutarate, Pyr: pyruvate, Gln: glutamine.

Since ROS regulates the OGDH and AMP-activated protein kinase (AMPK) [8,37,38], we evaluate the total-OGDH, phospho-, and total-AMPK levels at 4 h of treatment in BC cells. FRI-1 increases the phospho-AMPK levels, without changes in the OGDH levels in both BC cell lines (Figure 7). AMPK activation can promote cytoprotection as we previously described [29,31,39]. To prove this hypothesis, we treated BC cells with FRI-1 in presence of the AMPK inhibitor dorsomorphin (Dorso, 10 µM). The combination FRI-1 + Dorso significantly increased cell death at 48 h of treatment (Figure 8A), suggesting that FRI-1 induces a pro-survival AMPK signaling in BC cells.

### 3.6. FRI-1-Induced BC Cell Death Is Prevented by Dimethyl-aKG and Sensitized by Lipoic Acid or CPI-613 Combination

As FRI-1 produces elevated mtROS levels, we evaluate if FRI-1-induced cell death is mediated by redox imbalance that promotes TCA cycle disruption. We speculate that redox-sensible activities of PDH and OGDH, two 2-ketoacid dehydrogenases of the TCA cycle that has lipoic acid as a cofactor [28], may be affected. Using CPI-613 (150 µM), a dual inhibitor of PDH and OGDH activities (Figure 8H), at 48 h of treatment, we do not observe changes in the viability, however, the combination FRI-1 (25 µM) plus CPI-613 produced extensive death in both BC cell lines (Figure 8A,B). This combination promotes increased late apoptotic and necrotic subpopulations (Supplementary Information Figure S2A,B). Moreover, increased mitochondrial ROS levels were observed with CPI-613 plus FRI-1 combination (Figure 8C,D). To evaluate whether the redox-dependent regulation of OGDH may be affected by FRI-1, MCF7 cells were pre-incubated with the cofactor lipoic acid (5 mM), which enhances the OGDH activity as described [40], or were incubated with dimethyl-αKG (dm-αKG, 5 mM), a cell-permeable substrate of OGDH. As Figure 8E–G shows, FRI-1-induced cell death is prevented by dimethyl-αKG, and conversely, lipoic acid supplementation increases its cytotoxic effect. In MCF7 cells, no changes in the cell death are observed when FRI-1 is combined with antioxidant N-acetylcysteine (NAC) or dicumarol (Dic), an inhibitor of quinone metabolizing enzyme NAD(P)H quinone dehydrogenase 1 (NQO1) that mediates quinone reduction by NAD(P)H consumption and ROS production [41] (Supplementary Information Figure S3). Collectively, these results suggest that FRI-1 produces mitochondrial dysfunction through increased ROS production, affecting redox-dependent regulation of the TCA cycle and determining BC cell death.

## 4. Discussion

**Figure 9:**
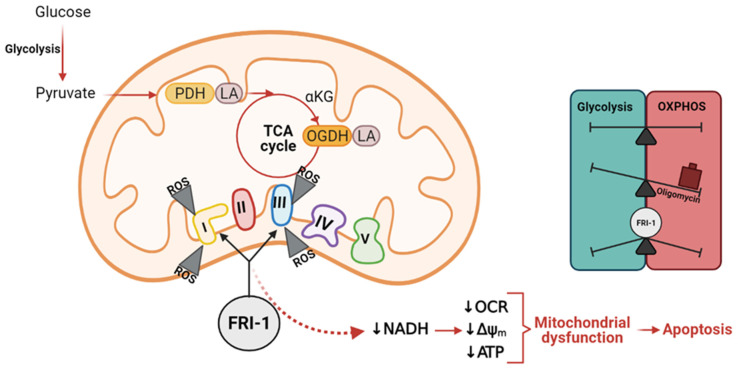
Mechanism of action proposed for FRI-1 compound.

Increased evidence indicates mitochondrial metabolism is essential for survival, metastasis, and chemoresistance in BC cells, becoming mitochondria in an attractive target for new anti-cancer compounds. Previously, FRI-1 was identified as a compound with a selective cytotoxic effect on cancer cell lines [3]; however, the mechanism of action remains still uncertain. In this work, we described that the electron transport chain is the main site of action of FRI-1, producing cell death by mitochondrial redox disruption (Figure 9).

Although OXPHOS inhibitors produce different anticancer effects [32,42,43], the most reported anticancer strategy is to promote apoptotic cell death by mitochondrial ROS production [30,42]. In this line, we describe that FRI-1 produces a burst of oxygen consumption in isolated mitochondria, which is partially prevented by ETC inhibitors. In intact BC cells, FRI-1 increases the basal mitochondrial OCR but blocks the maximal electron transport flux. These events are accompanied by Δψm drop and increased mitochondrial superoxide production. Consistent with a redox imbalance, the BC cells treated with FRI-1 exhibited reduced NADH and GSH/GSSG levels. Under these conditions of metabolic stress, it is usually produced AMPK-dependent metabolic remodeling toward glycolysis to maintain the ATP levels [36,44], however, FRI-1-induced mitochondrial dysfunction promoted AMPK phosphorylation, lacking metabolic shifts to glycolysis. Consistently, a reduction in intracellular ATP levels and increased apoptotic cell death in BC cells was observed. Interestingly, these results indicate that FRI-1 blocks the cancer cell’s escape to metabolic disruptions that are recognized for favoring survival, re-growth, and chemoresistance [45,46].

Although further studies are necessary to establish the mechanism involved in the inhibition of metabolic shifts by FRI-1, the increased production of mtROS may be relevant for inhibiting glycolysis. We observed that the FRI-1 analog with an eight carbon-length alkyl chain (FRI-3) exhibits different effects on ETC and mitochondrial bioenergetics compared to FRI-1. Interestingly, our results suggest that FRI-3 is a Complex I inhibitor that produces a metabolic shift toward glycolysis, maintaining the ATP levels and lacking the increase in mtROS levels observed for FRI-1. Moreover, increased glycolysis induced by oligomycin is not affected by FRI-3. It is recognized that acute but marked increase of intracellular ROS levels inhibits glycolysis by oxidation of Cys153 and Cys358 residues of glyceraldehyde 3-phosphate dehydrogenase (GAPDH) and pyruvate kinase M2 (PKM2), respectively [47]. This glycolytic inhibition promotes flux into the oxidative pentose phosphate pathway to produce NADPH and fuel cellular antioxidant systems for controlling the oxidative stress [48], however, FRI-1 produces a strong redox imbalance that is not restored by antioxidant defenses in BC cells.

Mitochondria control the redox homeostasis through Complex I and III activities in ETC, which is involved in supporting proliferative and metastatic signaling in cancer cells [21,49]. In conditions of increased mitochondrial redox stress, 2-ketoacid dehydrogenases of the TCA cycle, such as PDH and OGDH, are sensitive to oxidation, reducing its enzymatic activities. This occurs in the subunits that contain lipoic acid, an essential cofactor for participating in the stabilization and redox-dependent regulation of these multienzyme complexes [28]. Interestingly, reduction of OGDH activity may promote αKG accumulation, stabilizing hypoxia-inducible factor 1α (HIF1α) [50] which in turn governs gene expression-dependent metabolic adaptations that promote tumorigenesis [51], but also OGDH inhibition by small-molecule AA6 inhibits the metastasis in vivo [52]. Consistent with these contradictory events, different ROS levels produced by Complex I have anti- or pro-tumoral effects. ETC overload by an excess electron from the TCA cycle or partial ETC inhibition using low concentrations of rotenone (Complex I inhibitor) promote mitochondrial superoxide-dependent pro-metastatic phenotype in vitro and in vivo [18]. Conversely, full ETC inhibition is produced by high concentrations of rotenone block superoxide production, generating anticancer effects [18]. The grade of ETC inhibition is a determinant for mtROS-dependent anticancer actions [32]. Since α-KG, but not NAC supplementation, partially prevents the cytotoxic effects of FRI-1, and lipoic acid increases the cell death, OGDH inhibition emerges as an essential step for the anti-cancer effect of FRI-1.

## 5. Conclusions

FRI-1 was previously identified as a compound with selective cytotoxic effects on cancer cell lines [3]; however, its mechanism of action has not yet been elucidated. Our results suggest that the anticancer effects of FRI-1 involve inhibition of mitochondrial bioenergetics by redox disruption in BC cells. For this, a redox-cycling dependent mainly on Complexes I and III produce mitochondrial dysfunction with a notable increase of mitochondrial ROS production, which may inhibit the redox-sensible TCA cycle enzymes PDH and OGDH (Figure 9).

## Data Availability

The data is contained within the article or supplementary material.

## Supplementary Materials

The following are available online at https://www.mdpi.com/article/10.3390/antiox10101618/s1, Supplementary Figure S1. FRI-3 inhibits the mitochondrial respiration, producing a metabolic remodeling toward glycolysis and lacks effects on oligomycin-induced glycolysis in BC cells; Supplementary Figure S2. Cell death stages produced by FRI-1 + CPI-613 combination in BC cells; Supplementary Figure S3. Effect of antioxidant NAC and NQO1 inhibitor dicoumarol (Dic) on cell death induced by FRI-1 in MCF7 cells, Supplementary Figure S4. Uncropped Western blots: Relative to Figure 4, MCF7 and MDA-MB-231 breast cancer cells.
