# Supplementary material for: FRI-1 Is an Anti-Cancer Isoquinolinequinone That Inhibits the Mitochondrial Bioenergetics and Blocks Metabolic Shifts by Redox Disruption in Breast Cancer Cells
Source: Antioxidants (Basel). 2021 Oct 14;10(10):1618. doi: 10.3390/antiox10101618 (PMC8533268; doi:10.3390/antiox10101618)
Supplement: Supplementary file 1 [file antioxidants-10-01618-s001.zip › antioxidants-1385916-supplementary.pdf]

FRI-1 is an anti-cancer isoquinolinequinone that inhibits the mitochondrial bioenergetics and blocks metabolic shifts by redox disruption in breast cancer cells.

Miguel Córdova-Delgado, Sebastián Fuentes-Retamal, Charlotte Palominos, Camila López-Torres, Daniela Guzmán-Rivera, Oney Ramírez-Rodríguez, Ramiro Araya-Maturana and Félix A. Urra.

Supplementary information

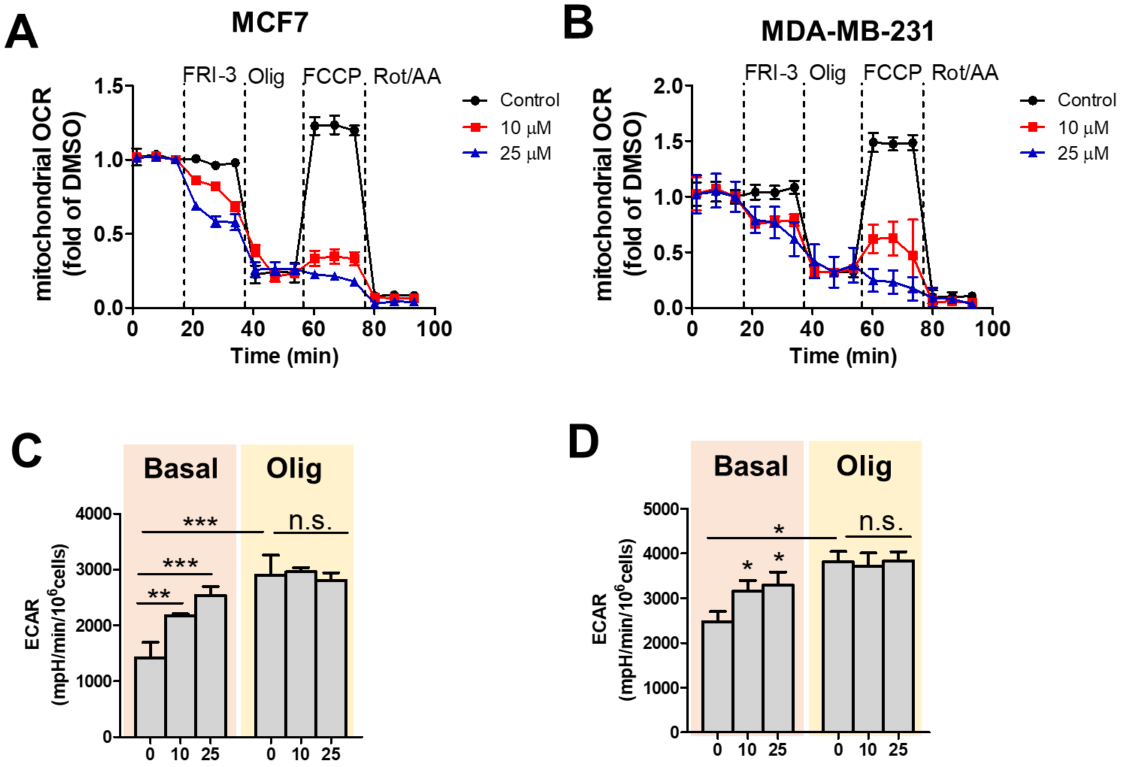

Supplementary Figure S1. FRI-3 inhibits the mitochondrial respiration, producing a metabolic remodeling toward glycolysis and lacks effects on oligomycin-induced glycolysis in BC cells. Data shown are the mean ± SD of three independent experiments. * $p < 0.05$, ** $p < 0.01$, *** $p < 0.001$, vs. control (DMSO). n.s.: not significant.

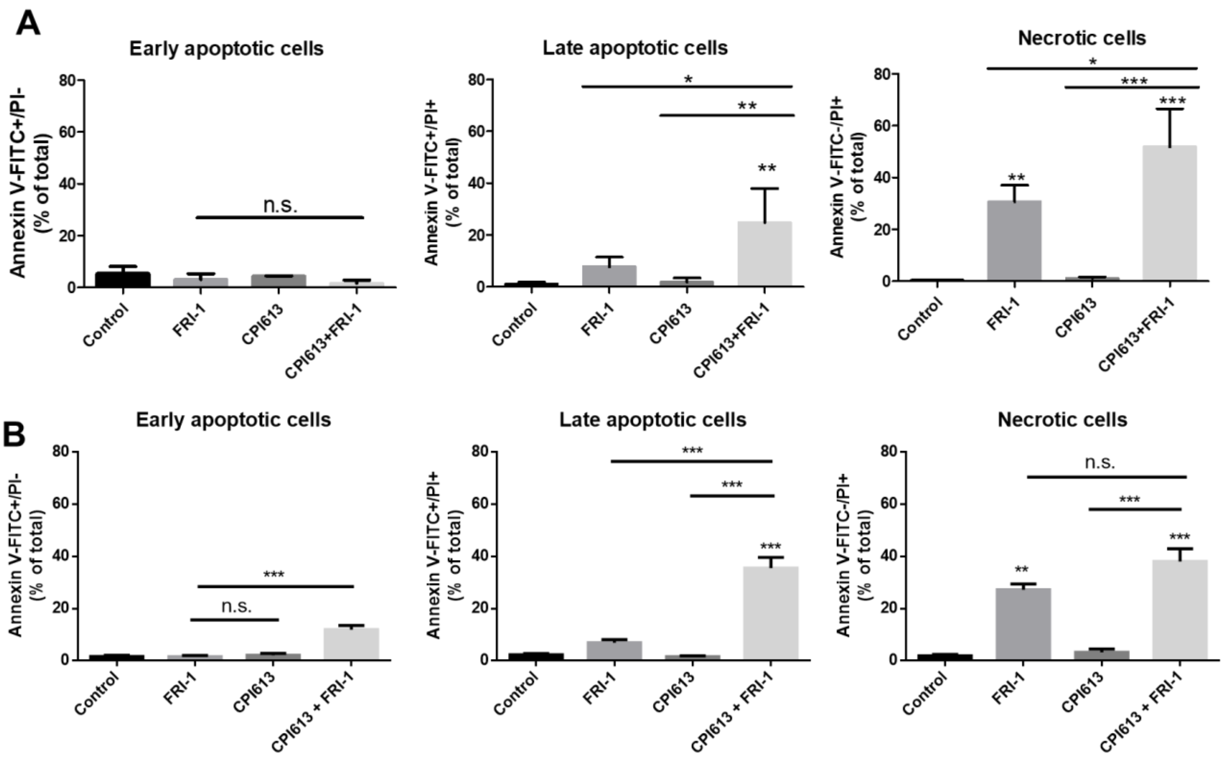

Supplementary Figure S2. Cell death stages produced by FRI-1 + CPI-613 combination in MCF7 (A) and MDA-MB-231 cells (B). Subpopulations for Annexin V-FITC and PI were evaluated at 48 h of treatment. Data shown are the mean ± SD of three independent experiments. * $p < 0.05$, ** $p < 0.01$, *** $p < 0.001$, vs. control (DMSO). n.s.: not significant.

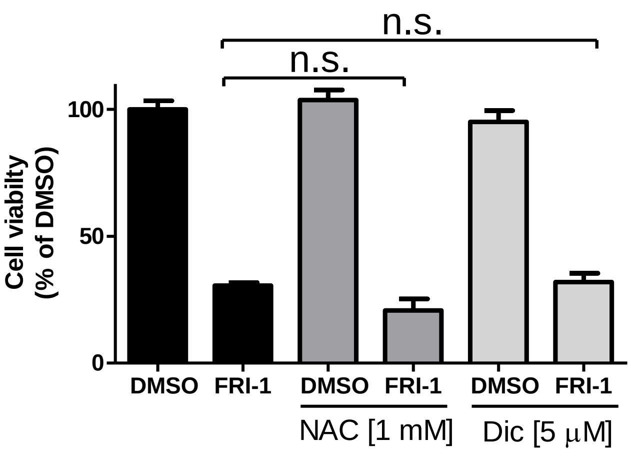

Supplementary Figure S3. Effect of antioxidant NAC and NQO1 inhibitor dicoumarol (Dic) on cell death induced by FRI-1 in MCF7 cells. Cells were incubated with NAC or Dic 1 h prior to the FRI-1 treatment (25 µM) and the viability was analyzed at 24 h. Data shown are the mean ± SD of three independent experiments. n.s.: not significant.

Uncropped Western blots

N1 – MCF7 cells

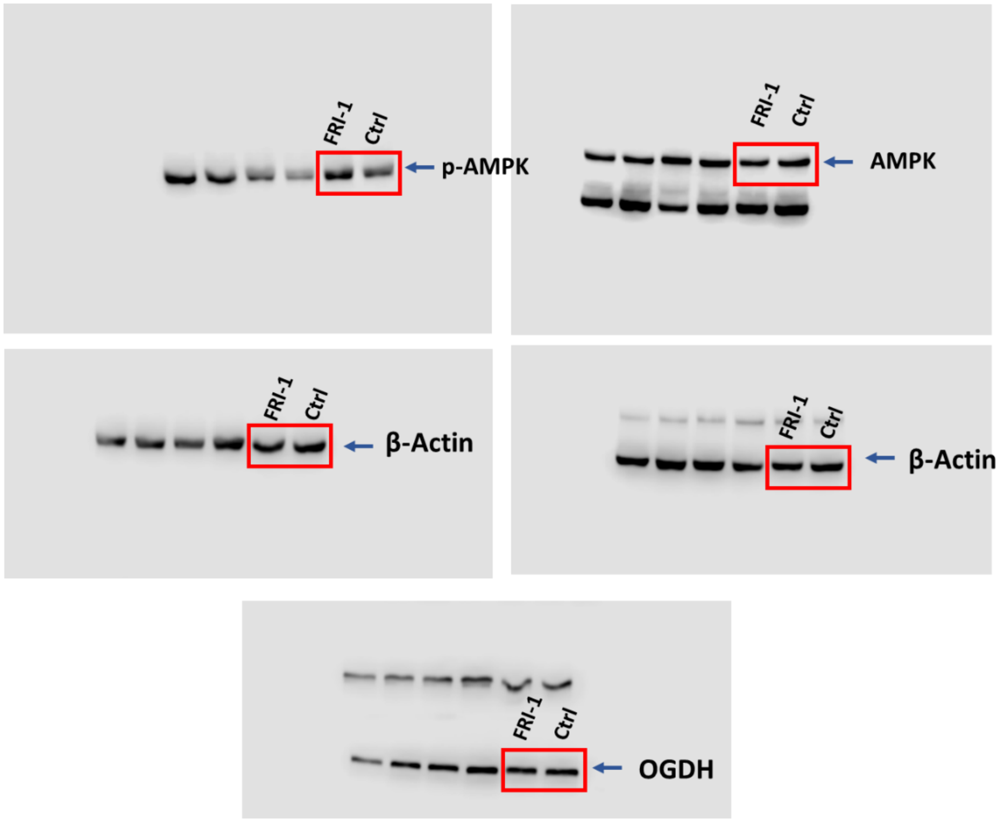

(Image used for representative Figure)

N2- MCF7 cells

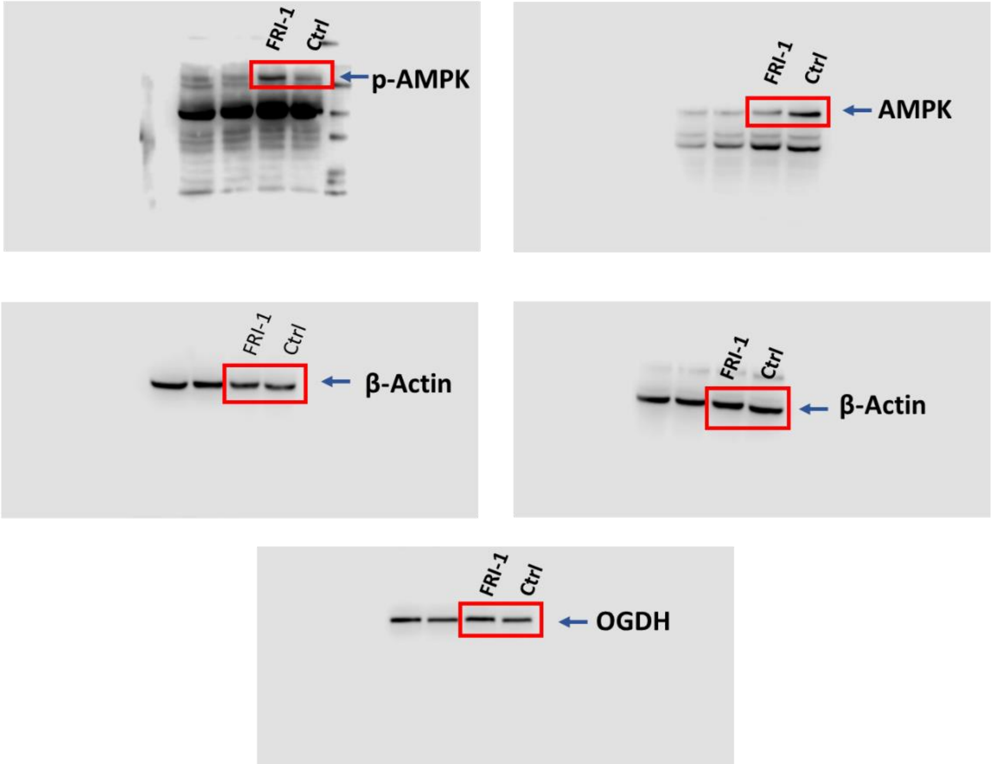

N3 – MCF7

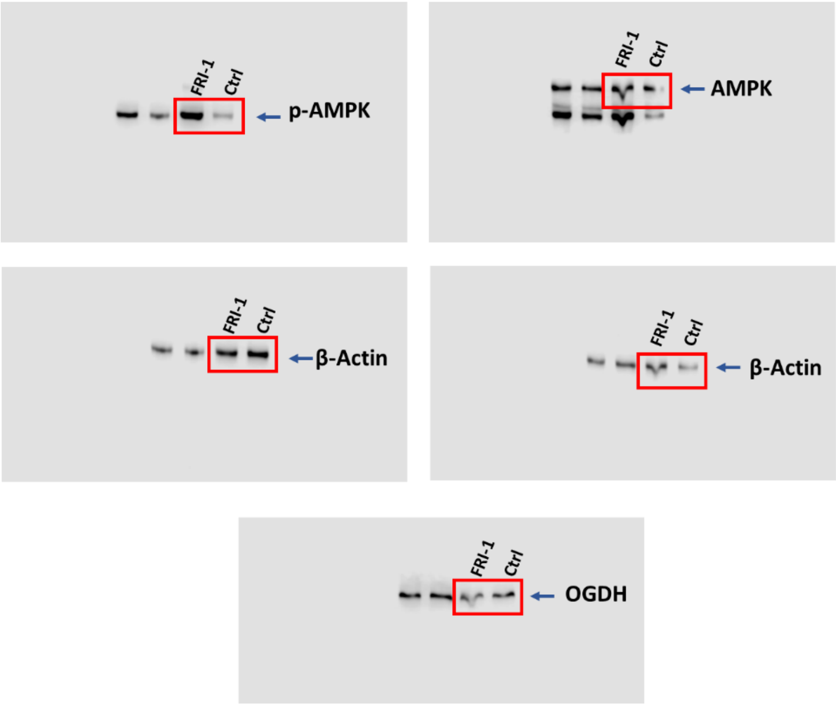

N1 – MDA-MB-231 cells

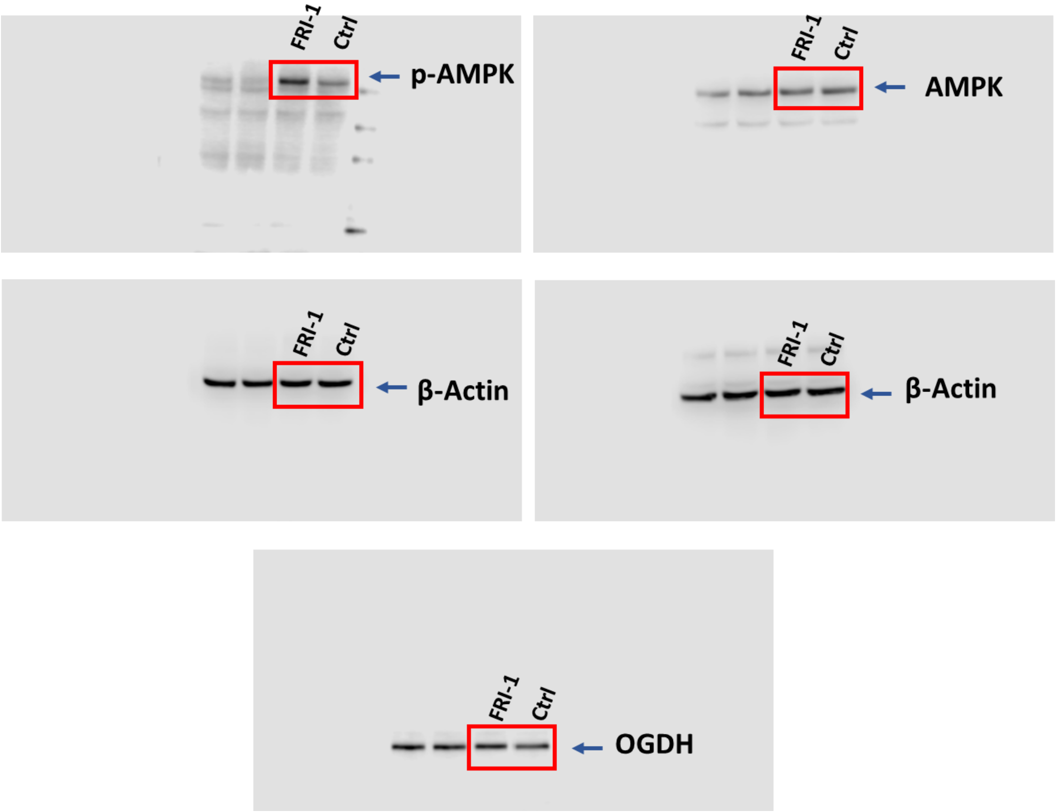

(Image used for representative Figure)

N2 and N3 – MDA-MB-231 cells

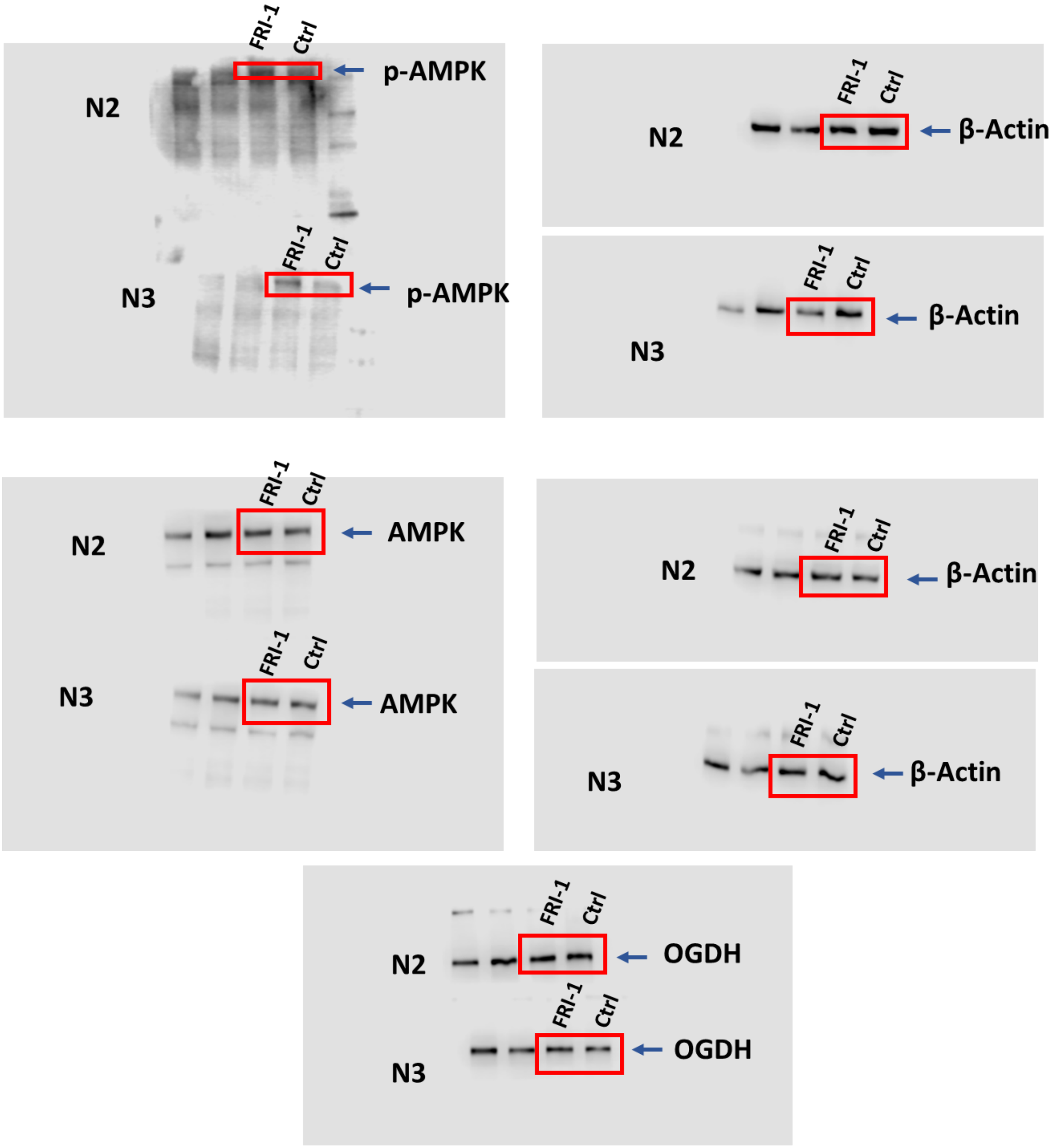

Supplementary Figure S4. Uncropped Western blots: Relative to Figure 4, MCF7 and MDA-MB-231 breast cancer cells.
